# Non-invasive prediction of lymph node involvement in prostate cancer via machine learning on whole-prostate MRI

**DOI:** 10.3389/fonc.2026.1823708

**Published:** 2026-05-11

**Authors:** Yun Luo, Bohao Liu, Peng Qin, Zhengxu Lin, Tianxin Lin, Yujie Wang

**Affiliations:** 1 Department of Urology, The First Affiliated Hospital of Xinjiang Medical University, Urumqi, China; 2 Department of Urology, The First People’s Hospital of Kashgar, Kashgar, China; 3 Xinjiang Medical University, Urumqi, China; 4 Department of Urology, The Third Affiliated Hospital of Sun Yat-sen University, Guangzhou, China; 5 Department of Urology, Sun Yat-sen Memorial Hospital of Sun Yat-sen University, Guangzhou, China

**Keywords:** deep learning, lymph node involvement, prediction model, prostate cancer, radiomics

## Abstract

**Background:**

Assessment of lymph node status is essential for guiding surgical decisions, prognosis evaluation, and recurrence risk estimation in prostate cancer (PCa). Therefore, this research seeks to establish and corroborate an integrated prediction model that leverages T2-weighted MRI scans to accurately identify lymph node involvement (LNI) in PCa patients.

**Methods:**

A retrospective cohort of 339 prostate cancer patients who underwent preoperative whole-prostate MRI followed by radical prostatectomy and extended pelvic lymph node dissection (ePLND) was evaluated. To non-invasively predict LNI, we developed a composite machine learning model integrating MRI-derived radiomics, deep learning features, and clinical parameters based on the 2012 Briganti nomogram. Model performance and clinical utility were assessed using the area under the receiver operating characteristic curve (AUC) and decision curve analysis (DCA).

**Results:**

The DRBN model demonstrated excellent predictive power in both the training and validation sets, with ROC-AUC scores of 0.963 and 0.920, and PR-AUC values of 0.950 and 0.921, correspondingly. Additionally, the DRBN model exhibited satisfactory calibration and clinical utility in both cohorts.

**Conclusion:**

Integrating clinical, radiomic, and deep learning features from whole-prostate MRI provides a feasible non-invasive approach for predicting LNI.

## Introduction

Prostate cancer (PCa) ranks as the second most frequently diagnosed malignancy and is the fifth leading cause of cancer mortality among men globally (1, 2). It is estimated that by 2040, new PCa cases will increase to 2.9 million, representing nearly a twofold rise compared to 2020 (3). As metastatic disease results in poorer quality of life, reduced survival, and limited treatment options (4), early identification and precise evaluation of metastasis remain imperative for patients with PCa.

Pelvic lymph node involvement (LNI) constitutes a key indicator of regional dissemination in PCa and commonly occurs prior to the distant metastasis (5). Approximately 12-15% of PCa patients experience LNI, which is associated with a significantly increased risk of recurrence and a poorer prognosis (6). Bernstein et al. demonstrated that PCa patients with pelvic LNI had a significantly increased risk of cancer-specific mortality (HR = 4.5) (7). Kim et al. reported that even patients with a single positive lymph node had a 5-year biochemical recurrence-free survival rate of only 19.1% (8). While the therapeutic benefit of extended pelvic lymph node dissection (ePLND) remains controversial, it is the gold standard for nodal staging in PCa (9, 10). Improved LNI prediction can help optimize staging-oriented surgical decision-making. Therefore, developing a non-invasive tool to preoperatively assess LNI risk is clinically imperative.

Magnetic resonance imaging (MRI), with its non-invasive nature and superior soft tissue resolution, has become a pivotal imaging modality for PCa, playing an irreplaceable role in clinical management (11, 12). Radiomics facilitates the extraction of high-dimensional quantitative features from medical images that surpass conventional visual assessment, and has been extensively utilized for the identification, staging, and prognostic evaluation of PCa (13–15). Traditional radiomics features offer good interpretability but are limited by issues such as feature instability, inconsistencies in data pre-processing, and insufficient generalizability (16). Compared to the former, convolutional neural networks (CNN) derived deep learning features can automatically learn more abstract and nonlinear representations, better capture spatial heterogeneity and the tumor microenvironment, and demonstrate stronger generalizability to diverse disease patterns (17). Therefore, combining radiomics features with deep learning features has the potential to leverage the complementary advantages of both approaches, thereby achieving improved predictive performance. In addition, compared to analyzing only the tumor lesion, evaluating the entire prostate gland—which encompasses lesions along with their surrounding microenvironment—can provide a more comprehensive understanding of the tumor, thereby capturing richer and more complete information about the disease (18).

In the present study, radiomics and deep learning techniques were employed to derive informative imaging features from the whole prostate volume on T2-weighted MRI, with the goal of establishing a non-invasive preoperative prediction model for LNI in PCa. This framework has the potential to assist urologists in personalized preoperative risk stratification.

## Materials and methods

### Patients

**Figure 1:**
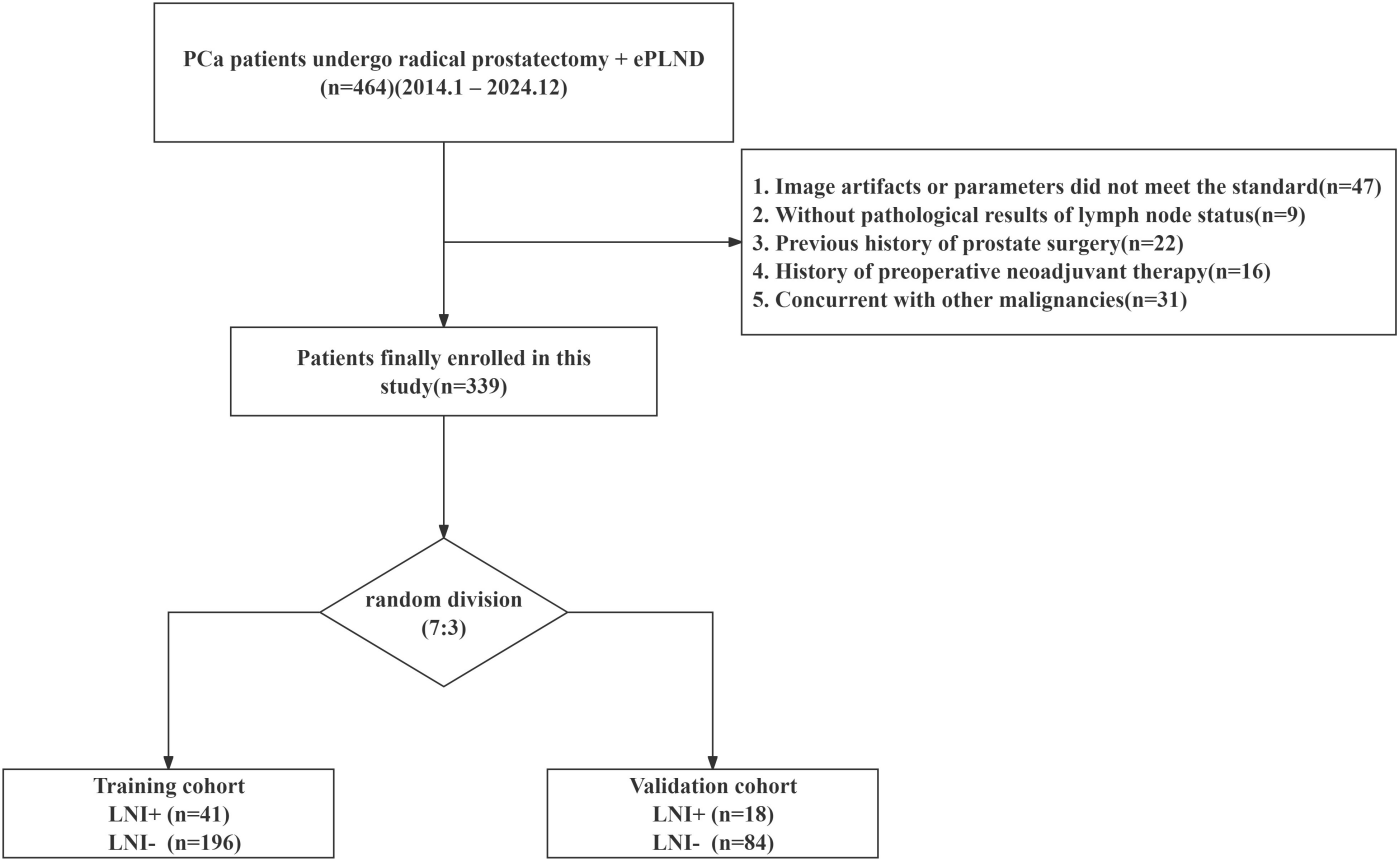
Flowchart of patient inclusion and grouping. PCa, prostate cancer, ePLND, extended pelvic lymph node dissection, LNI+, lymph node involvement–positive; LNI−, lymph node involvement–negative.

Patients who underwent prostate MRI followed by radical prostatectomy with ePLND for PCa at our institution between January 2014 and December 2024 were retrospectively included in this study. Clinical, imaging, and pathology data were accessed and extracted from the institutional databases and electronic medical records between April 2025 and June 2025. Exclusion criteria included (a) image artifacts or imaging parameters that did not meet the required standards; (b) absence of pathological results regarding lymph node status; (c) previous history of prostate surgery (detailed definition provided in the **Supplementary File**); (d) history of preoperative neoadjuvant therapy; and (e) concurrent diagnosis of other malignancies. The detailed flowchart of patient selection is presented in **Figure 1**. Given the retrospective design, informed consent was waived by the Ethics Committee of the Third Affiliated Hospital of Sun Yat-sen University (approval number: II2025-096-01). Ultimately, 339 subjects met the inclusion criteria and were enrolled, then randomly allocated to either the training or validation cohorts at a 7:3 ratio using stratified sampling based on LNI status.

### Image acquisition and segmentation

All imaging examinations were performed at a single center using 3.0-Tesla MR scanners (MAGNETOM Prisma, Siemens Healthcare, Germany). The imaging protocols were developed in accordance with the recommendations of PI-RADS version 2.1 (19). **Supplementary Table 1** contains comprehensive details of the scanning parameters.

The prostate gland was manually delineated as the region of interest (ROI) on axial T2-weighted MRI images, spanning from the apex to the base of the prostate, using 3D Slicer software (version 5.2.1). Two radiologists with 5 (reader1) and 15 (reader2) years of experience manually segmented the ROI, both of whom were blinded to the clinical and pathological information. To evaluate segmentation consistency, 30 patients were randomly selected, and both radiologists independently delineated ROIs for these cases. Reader1 then completed the segmentation of the remaining images one week later. The finalized delineations were saved as mask files in NRRD format.

### Radiomics features extraction

Radiomics features were extracted using the Pyradiomics library (version 3.0.1) running on Python (version 3.12.4). To ensure methodological consistency, feature extraction strictly complied with the recommendations established by the Image Biomarker Standardization Initiative (20). The features obtained included first-order statistics, shape-related descriptors, and various texture features, including gray-level co-occurrence matrix (GLCM), gray-level run length matrix (GLRLM), gray-level size zone matrix (GLSZM), gray-level dependence matrix (GLDM), and neighboring gray-tone difference matrix (NGTDM). Comprehensive information on these radiomic features is provided in the **Supplementary File**.

### Deep learning feature extraction

Each image was read and uniformly resized to 224 × 224 pixels using linear interpolation to standardize spatial dimensions across the dataset. The images were then converted from blue-green-red color space (BGR) to red-green-blue color space (RGB), and the pixel intensities were normalized to a mean and standard deviation of 0.5 for each channel to match the requirements of the deep learning model. Subsequently, we utilized the ResNet50 model pre-trained on ImageNet (http://www.image-net.org) as the backbone for feature extraction in the PyTorch environment. A custom module was employed to extract feature vectors from the global average pooling layer, ensuring that deep semantic representations of each image were captured. The details can be found in the **Supplementary Files**.

### Feature selection and model construction

A four-step protocol was adopted within the training set to achieve dimensionality reduction and robust selection of radiomics and deep learning features. Initially, 30 patients were randomly chosen for ROI segmentation and feature extraction, followed by assessment of feature stability and reproducibility through ICC calculation. Features with ICC above 0.6 were deemed sufficiently reproducible and retained for further processing. Second, the selected features were standardized using Z-score normalization. Third, group differences were assessed using either Student’s t-test or Mann–Whitney U-test, retaining features with an adjusted p-value less than 0.05. Fourth, least absolute shrinkage and selection operator (LASSO) regression was utilized to identify the most informative features with non-zero coefficients, with the penalty parameter optimized through 10-fold cross-validation. Finally, the features selected by LASSO were incorporated into seven machine learning algorithms, including logistic regression (LR), support vector machine (SVM), XGBoost, Naive Bayes (NB), decision tree (DT), random forest (RF), and k-nearest neighbors (KNN), to construct the radiomics and deep learning models. All feature selection procedures were strictly performed within the training cohort. To determine the optimal algorithm, a stratified 5-fold cross-validation (CV) was performed within the training cohort for each of the seven candidate models. The algorithm with the highest mean CV-AUC was selected as the final predictive model. Subsequently, the performance of the finalized model was evaluated in the independent internal validation cohort.

The clinical prediction model employed in this study was based on the 2012 Briganti nomogram (21), which integrates multiple established clinical variables for the prediction of LNI in PCa. The included predictors were serum PSA, clinical T-stage, biopsy Gleason score, and the percentage of positive biopsy cores. Consistent with the original nomogram, primary Gleason Grade (≤3, ≥4) and Secondary Gleason Grade (≤3, ≥4) were regrouped to facilitate analysis and model development. A logistic regression algorithm was implemented to construct the clinical model in accordance with the Briganti nomogram parameters.

A comprehensive DRBN model was subsequently constructed by integrating the clinical prediction model with radiomic and deep learning models, employing logistic regression. The development of the DRBN model was performed using the training cohort and further evaluated via five-fold cross-validation to ensure internal stability, while its performance and reliability were assessed in an independent internal validation cohort. Further methodological details regarding this procedure are available in the **Supplementary File**. All models used a 0.5 probability threshold for binary classification.

### Model evaluation

**Figure 2:**
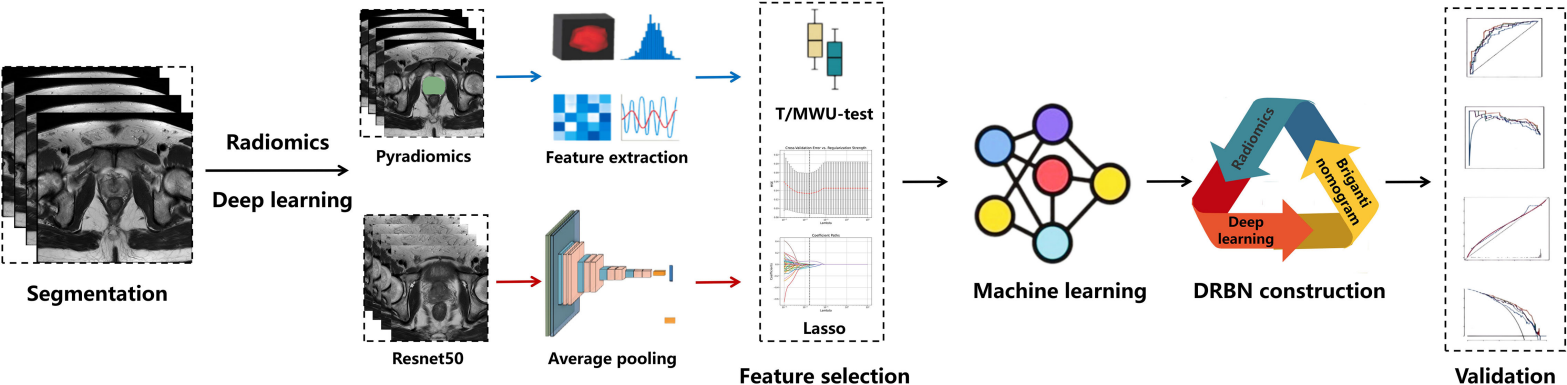
Workflow of the study. DRBN, Deep Learning–Radiomics–Briganti Nomogram model; T/MWU test, t-test and Mann-Whitney U test.

A comprehensive assessment of model performance was conducted by generating receiver operating characteristic (ROC) and precision-recall (PR) curves for all models in both the training and validation cohorts, with ROC-AUC and PR-AUC values reported accordingly. Furthermore, radar charts were employed to visually integrate and compare ROC-AUC, PR-AUC, sensitivity, specificity, positive predictive value (PPV), and negative predictive value (NPV) across models, enabling a visualization of their respective prediction capabilities. For the DRBN model, calibration curves along with Brier scores were used to assess model calibration and goodness of fit, while decision curve analysis (DCA) was performed to evaluate the clinical utility. Confusion matrices were presented for both cohorts to comprehensively summarize the classification results of the DRBN model. An overview of the steps involved in feature extraction, model building, and performance evaluation is illustrated in **Figure 2**. The contributions of the radiomics, deep learning, and clinical models to the integrated DRBN model were quantified using SHapley Additive exPlanations (SHAP) and visualized via SHAP beeswarm plots.

### Statistical analysis

Categorical variables were expressed as counts and percentages and compared using the Chi-square test or Yates’ continuity-corrected Chi-square test, as appropriate. Continuous variables were presented as mean ± standard deviation (SD) or as median with interquartile range (IQR) and compared using the Student’s t-test or Wilcoxon test, as appropriate. A two-tailed *P* value less than 0.05 was considered statistically significant. All statistical analyses were performed using R software (version 4.3.1) and Python (version 3.12.4).

## Results

### Patients’ characteristics

A total of 339 patients were included in the study, with 237 assigned to the training cohort (LNI+, n = 41) and 102 to the validation cohort (LNI+, n = 18). In the training cohort, statistically significant differences between groups were observed in several variables, including percentage of positive cores, cT, primary Gleason grade, and secondary Gleason grade (P < 0.05). However, no significant difference was detected in PSA (P > 0.05). In the validation cohort, significant inter-group differences were found in the percentage of positive cores, cT, and primary Gleason grade (P < 0.05), while PSA and secondary Gleason grade showed no significant differences (P > 0.05). Detailed clinical characteristics of enrolled patients are presented in **Table 1**.

**Table 1:** Clinical characteristics of enrolled patients.

Training cohort					Validation cohort				
Variables	LNI-	LNI+	P*	Comparison methods	Variables	LNI-	LNI+	P*	Comparison methods
	n = 196	n = 41				n = 84	n = 18		
PSA (ng/mL), median (IQR)	15.45 (8.58, 36.03)	20.91 (10.26, 48.89)	0.15	Wilcoxon test	PSA (ng/mL), median (IQR)	18.13 (9.65, 34.55)	34.54 (16.62, 88.73)	0.061	Wilcoxon test
Positive cores %, median (IQR)	0.33 (0.17, 0.77)	0.92 (0.58, 1)	<0.001	Wilcoxon test	Positive cores %, median (IQR)	0.5 (0.17, 0.80)	0.83 (0.59, 1)	<0.001	Wilcoxon test
cT, n (%)					cT, n(%)				
1	38 (19.4%)	1 (2.4%)			1	16 (19.0%)	1 (5.6%)		
2	118 (60.2%)	9 (22.0%)			2	43 (51.2%)	4 (22.2%)		
3	40 (20.4%)	31 (75.6%)	<0.001	Chi-square test	3	25 (29.8%)	13 (72.2%)	0.003	Yates’ correction
Primary Gleason Grade, n (%)					Primary Gleason Grade, n (%)				
0	10 (5.1%)	1 (2.4%)			0	5 (6.0%)	0 (0.0%)		
3	79 (40.3%)	1 (2.4%)			3	30 (35.7%)	1 (5.6%)		
4	86 (43.9%)	24 (58.5%)			4	43 (51.2%)	14 (77.8%)		
5	21 (10.7%)	15 (36.6%)	<0.001	Yates’ correction	5	6 (7.1%)	3 (16.7%)	0.031	Yates’ correction
Secondary Gleason Grade, n (%)					Secondary Gleason Grade, n (%)				
0	10 (5.1%)	1 (2.4%)			0	5 (6.0%)	0 (0.0%)		
3	90 (45.9%)	4 (9.8%)			3	29 (34.5%)	4 (22.2%)		
4	80 (40.8%)	15 (36.6%)			4	39 (46.4%)	10 (55.6%)		
5	16 (8.2%)	21 (51.2%)	<0.001	Yates’ correction	5	11 (13.1%)	4 (22.2%)	0.413	Yates’ correction
Primary Gleason Grade, n (%)					Primary Gleason Grade, n (%)				
≤3	89 (45.4%)	2 (4.9%)			≤3	35 (41.7%)	1 (5.6%)		
≥4	107 (54.6%)	39 (95.1%)	<0.001	Chi-square test	≥4	49 (58.3%)	17 (94.4%)	0.004	Chi-square test
Secondary Gleason Grade, n (%)					Secondary Gleason Grade, n (%)				
≤3	100 (51.0%)	5 (12.2%)			≤3	34 (40.5%)	4 (22.2%)		
≥4	96 (49.0%)	36 (87.8%)	<0.001	Chi-square test	≥4	50 (59.5%)	14 (77.8%)	0.146	Chi-square test

*Wilcoxon test; Chi-square test; Chi-square test(Yates’ correction); P values in bold are indicative of statistical significance (<0.05). IQR, Interquartile Range; PSA, Prostate Specific Antigen; cT, Clinical T stage.

### The feature extraction and selection

A total of 1,409 radiomics features and 2,048 deep learning (DL) features were extracted from axial T2-weighted MRI images. After removing features with poor reproducibility (ICCs < 0.6), 781 radiomics and 860 DL features were retained for further analysis using the Student’s t-test or Mann–Whitney U test. Subsequently, 125 radiomics and 106 DL features were selected for LASSO regression to optimize feature selection for machine learning model development. Ultimately, 9 radiomics features and 14 DL features were utilized to construct the radiomics and DL models, respectively (**Supplementary Figures 1A, B**, **2A, B**). The radiomics feature log-sigma-4-0-mm-3D_glrlm_HighGrayLevelRunEmphasis and the deep learning feature DL1661 demonstrated the highest coefficient weights within their respective models (**Supplementary Figures 1C**, **2C**).

### Construction and validation of models

**Figure 3:**
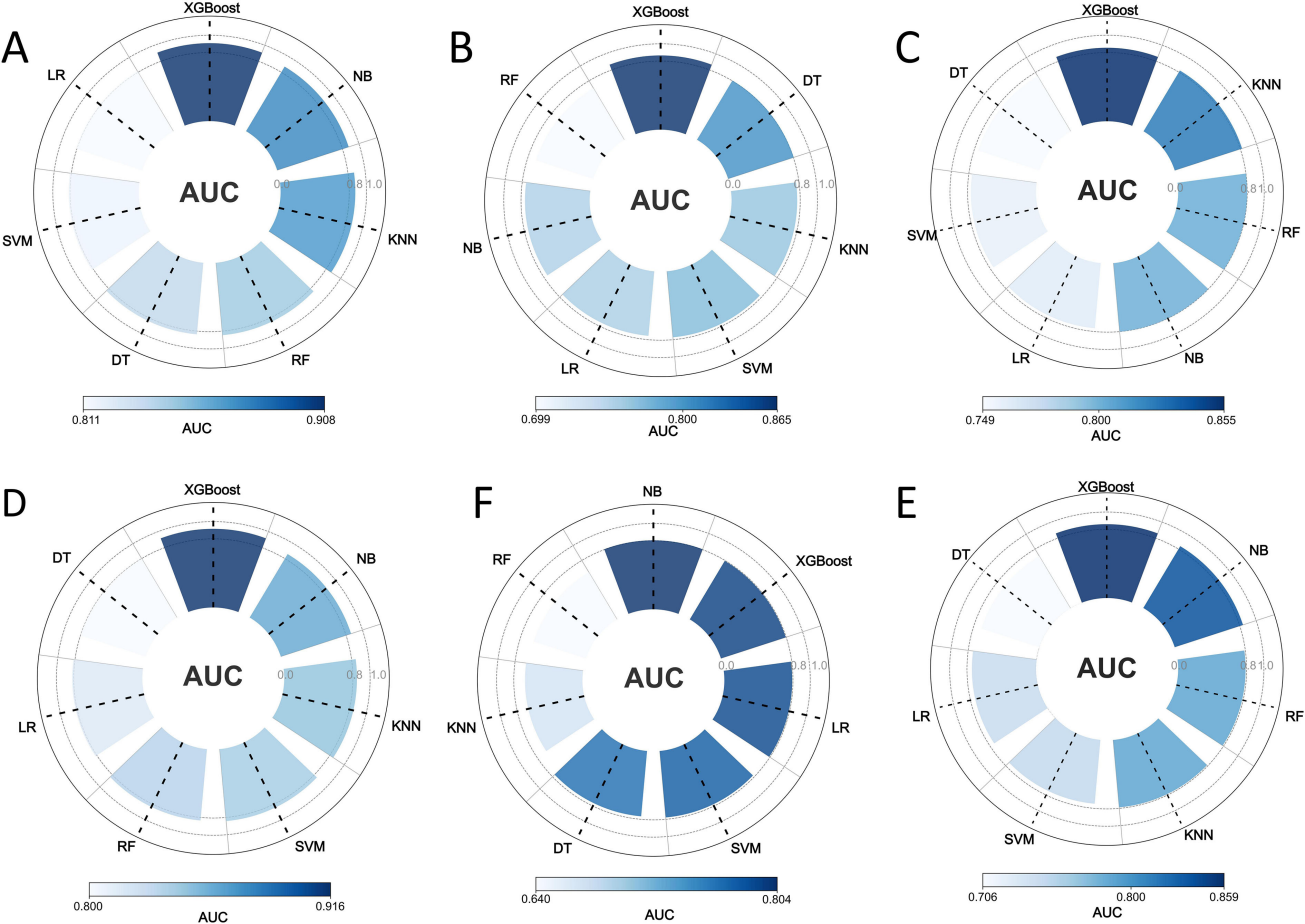
Comparison of radiomics and deep learning models using different machine learning algorithms in the training and validation cohorts. **(A–C)** Show the AUCs of radiomics models constructed using different machine learning algorithms: **(A)** AUCs in the training cohort, **(B)** AUCs in the validation cohort, and **(C)** mean CV-AUCs derived from stratified 5-fold cross-validation within the training cohort. **(D–F)** Show the AUCs of deep learning models constructed using the same machine learning algorithms: **(D)** AUCs in the training cohort, **(E)** AUCs in the validation cohort, and **(F)** mean CV-AUCs derived from stratified 5-fold cross-validation within the training cohort. AUCs, areas under the receiver operating characteristic curves, CV, cross-validation; LR, Logistic Regression; SVM, Support Vector Machine; RF, Random Forest; XGBoost, Extreme Gradient Boosting; KNN, K-Nearest Neighbors; NB, Naive Bayes; DT, Decision Tree.

Of the seven machine learning methods evaluated, XGBoost was selected to construct both the radiomics model (R-Model) and the deep learning model (DL-Model), based on its superior performance in the stratified 5-fold cross-validation within the training cohort. Specifically, the R-Model achieved a mean CV-AUC of 0.855 (95% CI: 0.813-0.896), with a corresponding training AUC of 0.908 and a validation AUC of 0.865. The DL-Model yielded a mean CV-AUC of 0.859 (95% CI: 0.775-0.942), with a training AUC of 0.916 and a validation AUC of 0.795 (**Figures 3A–F**). **Tables 2**, **3** summarize the diagnostic performance of all models, displaying AUCs for the training and validation cohorts alongside the internal mean CV-AUC. Additionally, a clinical predictive model for LNI was developed following the methodology of the Briganti nomogram (21).

**Table 2:** Diagnostic efficiency of different machine learning models with radiomics in the training and validation cohorts.

Model	AUC	95% CI	Accuracy	Sensitivity	Specificity	PPV	NPV	Cohort	Mean CV-AUC (95% CI)
XGBoost	0.908	(0.853-0.964)	0.839	0.898	0.78	0.803	0.885	Training cohort	0.855 (0.813-0.896)
XGBoost	0.865	(0.761-0.968)	0.765	0.76	0.769	0.76	0.769	Validation cohort	
DT	0.837	(0.764-0.910)	0.78	0.848	0.712	0.746	0.824	Training cohort	0.749 (0.661-0.837)
DT	0.804	(0.681-0.926)	0.765	0.8	0.731	0.741	0.792	Validation cohort	
KNN	0.87	(0.793-0.942)	0.759	0.806	0.729	0.658	0.854	Training cohort	0.824 (0.741-0.908)
KNN	0.765	(0.589-0.909)	0.706	0.733	0.684	0.647	0.765	Validation cohort	
NB	0.876	(0.793-0.944)	0.759	0.935	0.646	0.63	0.939	Training cohort	0.801 (0.742-0.860)
NB	0.754	(0.579-0.905)	0.8	0.676	0.579	0.6	0.786	Validation cohort	
SVM	0.816	(0.734-0.899)	0.7	0.707	0.699	0.33	0.919	Training cohort	0.758 (0.676-0.840)
SVM	0.772	(0.638-0.907)	0.667	0.778	0.643	0.318	0.931	Validation cohort	
LR	0.811	(0.728-0.894)	0.696	0.707	0.694	0.326	0.918	Training cohort	0.761 (0.666-0.856)
LR	0.755	(0.618-0.892)	0.618	0.722	0.595	0.277	0.909	Validation cohort	
RF	0.847	(0.812-0.883)	0.722	0.969	0.474	0.648	0.939	Training cohort	0.802 (0.685-0.920)
RF	0.699	(0.610-0.788)	0.578	0.778	0.536	0.264	0.918	Validation cohort	

AUC, area under the curve; CI, confidence interval; PPV, positive predictive value; NPV, negative predictive value, CV, cross-validation; Mean CV-AUC (95% CI), the mean AUC and its 95% confidence interval derived from 5-fold cross-validation in the training cohort; LR, Logistic Regression; SVM, Support Vector Machine; RF, Random Forest; XGBoost, Extreme Gradient Boosting; KNN, K-Nearest Neighbors; NB, Naive Bayes; DT, Decision Tree.

**Table 3:** Diagnostic efficiency of different machine learning models with deep learning features in the training and validation cohorts.

Model	AUC	95% CI	Accuracy	Sensitivity	Specificity	PPV	NPV	Cohort	Mean CV-AUC (95% CI)
XGBoost	0.916	(0.863-0.969)	0.847	0.915	0.78	0.806	0.902	Training cohort	0.859 (0.775-0.942)
XGBoost	0.795	(0.670-0.919)	0.804	0.84	0.769	0.778	0.833	Validation cohort	
NB	0.861	(0.771-0.935)	0.785	0.968	0.667	0.652	0.97	Training cohort	0.839 (0.796-0.882)
NB	0.804	(0.636-0.944)	0.735	0.8	0.684	0.667	0.812	Validation cohort	
SVM	0.842	(0.764-0.920)	0.759	0.854	0.74	0.407	0.96	Training cohort	0.746 (0.628-0.865)
SVM	0.778	(0.645-0.912)	0.667	0.889	0.619	0.333	0.963	Validation cohort	
LR	0.816	(0.734-0.899)	0.726	0.756	0.719	0.36	0.934	Training cohort	0.743 (0.628-0.857)
LR	0.791	(0.660-0.922)	0.676	0.722	0.667	0.317	0.918	Validation cohort	
DT	0.8	(0.719-0.880)	0.703	0.814	0.593	0.667	0.761	Training cohort	0.706 (0.636-0.777)
DT	0.765	(0.633-0.897)	0.745	0.72	0.769	0.75	0.741	Validation cohort	
KNN	0.847	(0.763-0.927)	0.747	0.839	0.688	0.634	0.868	Training cohort	0.788 (0.725-0.852)
KNN	0.67	(0.476-0.844)	0.647	0.6	0.684	0.6	0.684	Validation cohort	
RF	0.835	(0.798-0.872)	0.681	0.964	0.398	0.616	0.918	Training cohort	0.788 (0.744-0.832)
RF	0.64	(0.547-0.734)	0.637	0.611	0.643	0.268	0.885	Validation cohort	

AUC, area under the curve; CI, confidence interval; PPV, positive predictive value; NPV, negative predictive value, CV, cross-validation; Mean CV-AUC (95% CI), the mean AUC and its 95% confidence interval derived from 5-fold cross-validation in the training cohort; LR, Logistic Regression; SVM, Support Vector Machine; RF, Random Forest; XGBoost, Extreme Gradient Boosting; KNN, K-Nearest Neighbors; NB, Naive Bayes; DT, Decision Tree.

**Figure 4:**
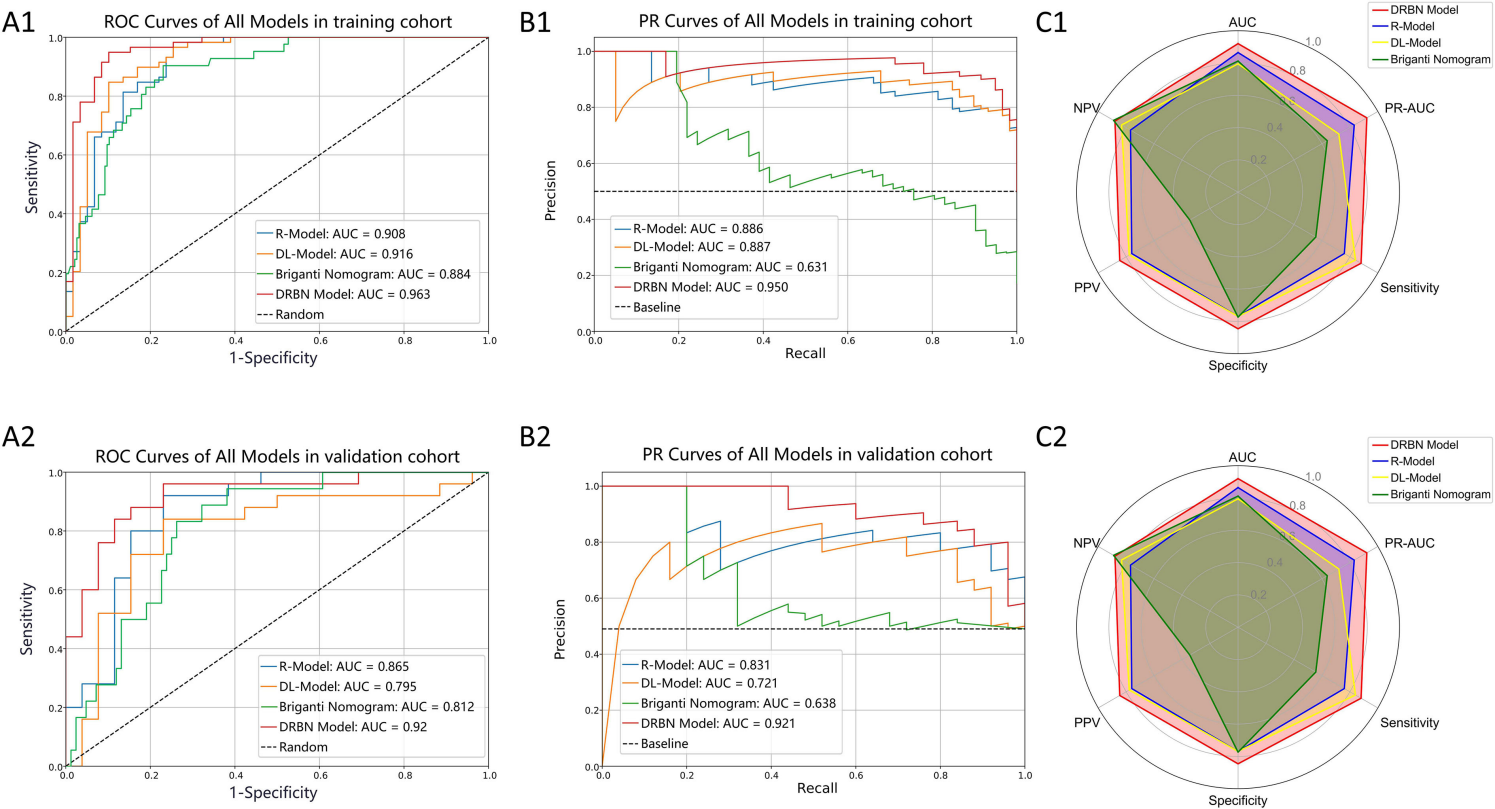
Performance evaluation of different models in the training and validation cohorts. **(A1, A2)** receiver operating characteristic (ROC) curves of all models in the training cohort **(A1)** and the validation cohort **(A2)**. **(B1, B2)** precision-recall (PR) curves of all models in the training cohort **(B1)** and the validation cohort **(B2)**. **(C1, C2)** radar charts displaying model performance across multiple metrics (AUC, PR-AUC, sensitivity, specificity, PPV, and NPV) in the training **(C1)** and validation **(C2)** cohorts. The DRBN model demonstrates superior discriminative performance (highest AUC and PR-AUC) in both cohorts compared to the R-model, DL-model, and Briganti nomogram. DRBN Model, Deep Learning–Radiomics–Briganti Nomogram model; R-Model, Radiomics Model; DL-Model, Deep Learning Model; PPV, positive predictive value; NPV, negative predictive value; AUC, area under the curve.

Four predictive models were developed for assessing LNI risk in patients with PCa: a clinical model, the R-Model, the DL-Model, and an integrated model (DRBN). **Table 4** outlines the predictive performance of all constructed models. As shown by the ROC and PR curves (**Figures 4A, B**), the DRBN model demonstrated superior predictive ability for LNI compared to the individual models. Specifically, the DRBN model achieved the highest ROC-AUC values in both the training cohort (AUC = 0.963, 95% CI: 0.927–0.998) and validation cohort (AUC = 0.920, 95% CI: 0.840–1.000). This advantage is further highlighted by the radar chart, which shows that the DRBN model excels across most relevant performance indicators (**Figure 4C**). Furthermore, internal stratified five-fold cross-validation yielded a mean AUC of 0.912 (95% CI: 0.871-0.953), with the consistent performance across resampling iterations underscoring the robustness and reliability of the framework (**Supplementary Figure 3**).

**Table 4:** Comparative performance of different models in the training and validation cohorts.

Cohort	Model	AUC	95% CI	PR-AUC	Accuracy	Sensitivity	Specificity	PPV	NPV
Training cohort	DRBN Model	0.963	(0.927-0.998)	0.95	0.907	0.915	0.898	0.9	0.914
	R-Model	0.908	(0.853-0.964)	0.886	0.839	0.898	0.780	0.803	0.885
	DL-Model	0.916	(0.863-0.969)	0.887	0.847	0.915	0.780	0.806	0.902
	Briganti Nomogram	0.884	(0.832-0.928)	0.631	0.831	0.463	0.908	0.514	0.890
Validation cohort	DRBN Model	0.92	(0.840-1.000)	0.921	0.863	0.88	0.846	0.846	0.88
	R-Model	0.865	(0.761-0.968)	0.831	0.765	0.760	0.769	0.760	0.769
	DL-Model	0.795	(0.670-0.919)	0.721	0.804	0.840	0.769	0.778	0.833
	Briganti Nomogram	0.812	(0.706-0.892)	0.638	0.735	0.556	0.774	0.345	0.890

AUC, area under the curve; CI, confidence interval; PR-AUC, precision-recall area under the curve; PPV, positive predictive value; NPV, negative predictive value; DRBN Model, Deep Learning–Radiomics–Briganti Nomogram model; R-Model, radiomics model; DL-Model, deep learning model; LR, Logistic Regression; SVM, Support Vector Machine; RF, Random Forest; XGboost, Extreme Gradient Boosting; KNN, K-Nearest Neighbors; NB, Naive Bayes; DT, Decision Tree.

**Figure 5:**
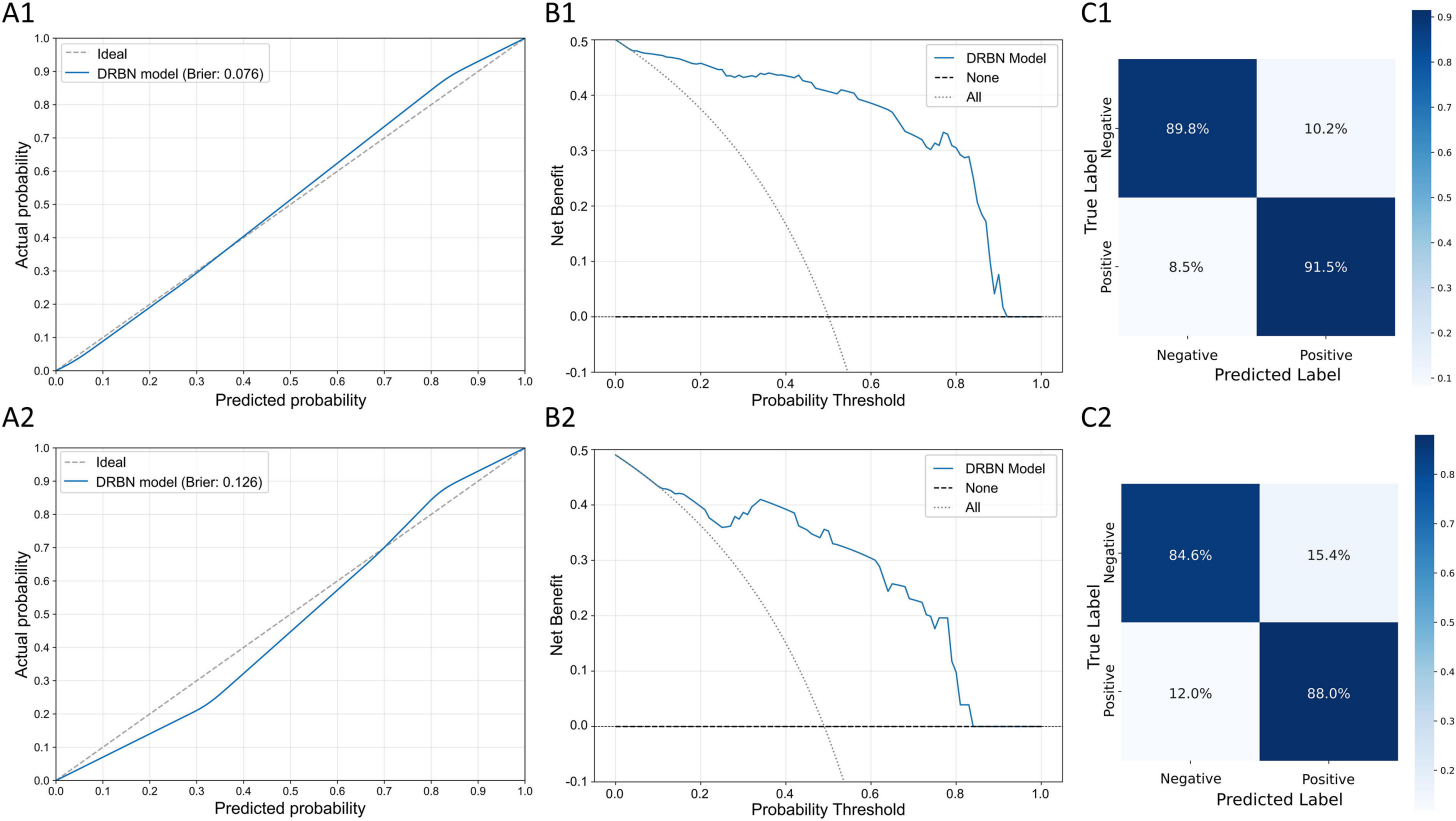
Calibration, decision curve analysis, and confusion matrix of the DRBN model in the training and validation cohorts. **(A1, A2)** Calibration curves for the DRBN model in the training **(A1)** and validation **(A2)** cohorts. The calibration curves compare the predicted probability with the actual probability, showing good agreement with the ideal line. Brier scores are indicated for each cohort. **(B1, B2)** DCA for the DRBN model in the training **(B1)** and validation **(B2)** cohorts. The net benefit is plotted against a range of probability thresholds, demonstrating the clinical usefulness of the model compared to the “None” and “All” strategies. **(C1, C2)** Confusion matrices for the DRBN model in the training **(C1)** and validation **(C2)** cohorts. The matrices present the proportion of true positive, false positive, true negative, and false negative classifications, indicating strong predictive performance in both cohorts. DRBN Model, Deep Learning–Radiomics–Briganti Nomogram model; DCA, Decision curve analysis.

With respect to calibration, the DRBN model exhibited low Brier scores in the training (0.076) and validation (0.126) cohorts, indicating satisfactory calibration, as visualized in the calibration curves (**Figure 5A**). Decision curve analysis further confirmed that the DRBN model provides a high net clinical benefit in both cohorts (**Figure 5B**). Moreover, the confusion matrices (**Figure 5C**) illustrate the classification performance of the DRBN model. In the training cohort, the model correctly identified 91.5% of positive cases and 89.8% of negative cases. In the validation cohort, the model achieved true positive and true negative rates of 88.0% and 84.6%. The results of the SHAP analysis indicated that the average SHAP value for the R-Model was the highest, followed by the DL-Model and the clinical model. This trend is clearly illustrated in the SHAP beeswarm plot (**Supplementary Figure 4**).

## Discussion

Drawing on retrospective data from 339 PCa patients, this study constructed and validated multiple predictive models for LNI. All models demonstrated acceptable predictive accuracy, while the DRBN model notably surpassed the remaining models in both cohorts, attaining the highest ROC-AUC values of 0.963 and 0.920 in the training and validation sets, respectively. To mitigate the impact of class imbalance and enhance the reliability of our findings, PR curves were also plotted as a supplementary evaluation. These analyses confirmed that the DRBN model maintained superior discriminatory power, yielding PR-AUC values of 0.950 and 0.921 for the training and validation cohorts, respectively. As an integrated approach combining clinical variables, radiomics features, and deep learning features, the DRBN model not only showed promising discrimination and calibration in our cohort but also suggested potential clinical utility as a supportive tool for risk stratification.

ePLND represents the gold-standard method for assessing LNI in PCa patients (22, 23). However, determining which patients genuinely require ePLND remains a significant challenge in clinical practice. Studies have demonstrated that approximately 47%-65.5% of ePLND procedures performed are unnecessary (24–26). These unwarranted ePLND procedures may lead to a spectrum of adverse outcomes, including lymphocele formation, deep vein thrombosis, neurovascular injury, and pelvic organ damage (27, 28). In this context, developing accurate preoperative noninvasive models to predict LNI is imperative. Such tools can help clinicians refine staging-oriented surgical decision-making and facilitate more personalized management for PCa patients.

Over the past several decades, predictive models for LNI in PCa have undergone extensive development. Among clinical nomograms, the Memorial Sloan Kettering Cancer Center (MSKCC) nomogram and Briganti nomogram have been widely implemented in clinical practice. These models primarily incorporate clinical and pathological variables. However, their predictive accuracy remains limited. In a multicenter study conducted by Meijer et al., the AUCs for the Briganti 2017, MSKCC, and Briganti 2019 nomograms were reported as 0.70, 0.71 and 0.76, respectively (29). Pierros et al. demonstrated that even when applying an optimal cut-off value of 7%, approximately 70% of patients undergoing ePLND ultimately had negative pathological lymph node findings (30). The 2012 Briganti nomogram was utilized due to its extensive validation across diverse populations (21, 27, 31). This choice ensures data consistency across our 2014–2024 cohort and minimizes chronological bias from the non-standardized documentation of newer parameters in earlier years, providing a stable baseline to evaluate the predictive performance of our AI-driven DRBN model.

Many studies have applied MRI-based radiomics features to predict LNI in PCa patients (14, 32–34). On one hand, most studies rely on PyRadiomics for radiomic feature extraction. Despite its prevalence, PyRadiomics focuses on traditional features (shape, intensity, texture), potentially missing higher-order and abstract information in medical images (35, 36). On the other hand, most radiomics studies focus on tumor or lymph node ROI, posing challenges for clinicians without specialized prostate MRI training (37). Studies indicate that even experienced radiologists show substantial inter-observer variability and occasional segmentation errors during manual delineation (38, 39). These limitations hinder the clinical applicability of such models.

Compared to traditional radiomic features, CNN derived deep learning features have notable advantages. They automatically learn more abstract and nonlinear representations, overcoming the limits of handcrafted features (16, 40). By integrating information across scales and hierarchical levels, CNN better capture spatial heterogeneity and tumor microenvironment, while their data-driven nature enables strong generalization to diverse disease patterns (17, 41). An increasing number of studies have utilized CNN to automatically extract deep learning features from medical images for predicting LNI in malignant diseases (42–45). These approaches have achieved high accuracy and AUC values, with diagnostic performance comparable to that of experienced radiologists. Moreover, combining deep learning features with traditional radiomic features in one model further enhances predictive performance and robustness. Lin et al. found that integrating deep learning, radiomics, and clinical data improved classification accuracy and generalizability for lung nodule diagnosis (46). Similarly, Bestetti et al. and Zhang et al. showed that the combined approach achieved higher AUC than either method alone, leveraging both the phenotypic sensitivity of deep learning and the interpretability of radiomics (47, 48). In our study, we extracted deep learning features using a CNN-based ResNet50 model, a 50-layer deep residual network composed of stacked residual blocks with identity shortcut connections. These shortcuts enable efficient gradient flow and help prevent vanishing or exploding gradients during deep network training (49), allowing ResNet50 to capture more complex feature representations. Ultimately, by integrating the selected deep learning features with radiomic features and clinical variables, our DRBN model achieved satisfactory predictive performance for LNI in PCa.

PCa demonstrates remarkable heterogeneity with characteristic multi-lesioned distribution patterns (50). Early-stage or diffusely infiltrating PCa present significant challenges for ROI delineation due to their occult nature and indistinct margins (39). Previous radiomics approaches that exclusively analyzed conspicuous lesions as ROI potentially overlooked clinically relevant microlesions and critical tissue-level alterations. In contrast, utilizing the entire prostate region as an ROI encompasses information from all cancer lesions and their tumor microenvironment, capturing more comprehensive pathological information. Multiple studies have successfully implemented whole-prostate ROI delineation strategies for PCa prediction, consistently demonstrating superior predictive performance and methodological stability across various clinical contexts (13, 15, 18, 51). Additionally, numerous studies have demonstrated that whole-prostate ROI exhibits superior robustness and reproducibility, with this simplified approach yielding more standardized outcomes (39, 51). Consistent with established articles in prostate radiomics research (52, 53), patients with a prior history of prostate surgery were excluded to mitigate potential interference from postoperative anatomical distortions or imaging artifacts on the fidelity of quantitative feature extraction, thereby confining to a treatment-naive population. In this study, we selected the entire prostate region as our ROI through axial T2-weighted MRI sequences, which is consistent with clinical workflow. Compared to complex lesion-specific segmentation, this approach has lower technical requirements, which may facilitate future clinical application of the model.

Objectively, our study presents several limitations. First, this investigation was a retrospective analysis conducted on 339 patients from a single medical center. Thus, our findings require further validation in larger cohorts across various medical centers and ethnicities, with future prospective trials needed to define its optimal clinical thresholds and real-world utility in surgical decision-making. Second, while the 2012 Briganti nomogram ensured data consistency across our cohort, we acknowledge that it does not incorporate contemporary modalities such as PSMA-PET or updated nomograms, which will be addressed in future prospective evaluations to further refine the DRBN model. Third, building the DRBN requires several steps, as explained in the methods section. Therefore, to promote broader adoption in future clinical practice, an automated and clinician-friendly software will be necessary to streamline access to DRBN for clinicians.

## Conclusion

The DRBN model, combining clinical factors, radiomics features, and deep learning signatures, exhibited favorable internal predictive performance for LNI in PCa. These preliminary findings remain exploratory and require multicenter external validation.

## Data Availability

The original contributions presented in the study are included in the article/**Supplementary Material**. Further inquiries can be directed to the corresponding authors.
