# Supplementary material for: Non-invasive prediction of lymph node involvement in prostate cancer via machine learning on whole-prostate MRI
Source: Front Oncol. 2026 May 11;16:1823708. doi: 10.3389/fonc.2026.1823708 (PMC13199099; doi:10.3389/fonc.2026.1823708)
Supplement: Supplementary file 1 [file DataSheet1.docx]

**Supplementary Material for**

**Non-invasive Prediction of Lymph Node Involvement in Prostate Cancer via Machine Learning on Whole-Prostate MRI**

**Supplement Methods**

Supplement I. Radiomics features

Supplement II. Deep learning features

Supplement III. Model construction (Machine Learning Algorithms)

Supplement IV. Definition of Prior Prostate Surgery

**Supplement Figures and Tables**

Supplement Figure 1. LASSO regression for radiomics feature selection.

Supplement Figure 2. LASSO regression for selection of deep learning features.

Supplement Figure 3. Five-fold cross-validation of DRBN in the training cohort

Supplement Figure 4. SHAP beeswarm plots for the DRBN model.

Supplement Table 1. Main Sequence Parameters of Prostate MRI Scans.

1. **Radiomics features**

B-Spline interpolation was employed for resampling, ensuring that all images were standardized to an isotropic voxel size of $1.0 \times1.0 \times1.0 mm$. From each delineated region of interest (ROI), a total of 1,409 quantitative imaging features were extracted. These comprised 14 shape descriptors, 18 first-order statistical metrics, 24 gray-level co-occurrence matrix (GLCM) features, 16 gray-level run length matrix (GLRLM) characteristics, 16 gray-level size zone matrix (GLSZM) features, 14 gray-level dependence matrix (GLDM) parameters, and 5 neighboring gray-tone difference matrix (NGTDM) indices. Additionally, 1,302 supplementary features were derived from images processed using a variety of filters, including wavelet transformations, Laplacian of Gaussian (LoG), exponential, square, square root, logarithmic, and gradient filters. Feature extraction was uniformly conducted on the segmented ROIs across all imaging datasets.

1. **Deep learning features**

To extract deep learning features from the ROI, we utilized a ResNet-50 convolutional neural network pretrained on the ImageNet dataset as the backbone, with all model weights initialized accordingly to benefit from comprehensive image representation capability. A custom feature extractor was designed by retaining all layers up to and including the average pooling layer, allowing extraction of a 2048-dimensional feature vector from each image. For image preprocessing, all 2D ROI tiles were resized to 224×224 pixels and normalized with a mean and standard deviation of (0.5, 0.5, 0.5) to match the ResNet-50 input specifications. After feature extraction, vectors from images belonging to the same patient (i.e., subject or ROI) were averaged to obtain a robust representative feature. These aggregated features were exported in Excel format for further analysis. A fixed random seed of 1314 was set to ensure reproducibility throughout the process.

1. **Model construction (Machine Learning Algorithms)**

To identify the best-performing radiomics and deep learning models, seven different machine learning algorithms were evaluated. All patients were randomly divided into training and validation cohorts at a 7:3 ratio. The DRBN model was constructed by integrating the predicted probabilities from the radiomics, deep learning, and clinical models using logistic regression in Python’s scikit-learn library.

1. Support Vector Machine (SVM)

SVM is a supervised learning algorithm commonly applied to classification and regression tasks. Its core principle is to identify an optimal hyperplane within an N-dimensional feature space that can effectively separate data points from different classes. Points located on opposing sides of the hyperplane are assigned to different categories. Additionally, SVM seeks to maximize the distance, or margin, between the data points and the separating hyperplane, thereby enhancing model robustness and generalization ability(Cortes and Vapnik 1995).

1. Naive Bayes (NB)

NB classifiers represent a family of simple probabilistic models that are based on Bayes’ theorem, assuming that all features are mutually independent. This strong independence assumption allows for efficient computation and is a key characteristic of the Naive Bayes approach(Schonlau 2023).

1. XGBoost

XGBoost is a powerful and versatile machine learning algorithm based on the gradient boosting framework, primarily utilizing decision tree ensembles. Distinguished by its support for parallelized tree construction, XGBoost can efficiently handle large-scale data and deliver high predictive accuracy across a wide range of data science applications. In addition to tree-based learning, it also includes linear model solvers, further enhancing its capabilities in processing structured data(Chen and Guestrin 2016).

1. Logistic Regression (LR)
   LR is a widely used statistical method for modeling binary or categorical outcome variables. It estimates the probability that a given input belongs to a particular class using the logistic (sigmoid) function, and parameters are typically obtained via maximum likelihood estimation. Logistic regression is valued for its simplicity, interpretability, and effectiveness in various fields such as medicine and social sciences(Bewick, et al. 2005).
2. Decision Tree (DT)
   DT is a non-parametric supervised learning algorithm typically used for classification and regression. It uses a tree-like model of decisions to split data into subsets based on feature values, making a sequence of decisions from root to leaf. Popular algorithms include CART and C4.5. Decision trees are easy to interpret but may be susceptible to overfitting(Song and Lu 2015).
3. Random Forest (RF)
   RF is an ensemble learning method that constructs multiple decision trees and merges their predictions to improve generalization and accuracy. Each tree in a random forest is built on a random subset of the data and features, reducing overfitting compared to single decision trees. Random forest is widely used due to its robustness and strong predictive performance(Breiman 2001).
4. k-Nearest Neighbors (KNN)
   The KNN is a simple, non-parametric method used for classification and regression. For a given sample, KNN assigns the label most common among its k closest neighbors in the feature space. It is easy to implement and does not make assumptions on data distribution, but can be computationally intensive on large datasets(Zhang 2016).

For each of the seven machine learning models, hyperparameters were predefined based on established literature and empirical conventions. Model performance was evaluated using 5-fold cross-validation within the training cohort, with AUC as the primary optimization metric to ensure generalizability. Final models were subsequently assessed on the independent validation cohort

1. **Definition of Prior Prostate Surgery**

“Previous history of prostate surgery” in this study was defined as any historical surgical or interventional procedure that alters the anatomical integrity or imaging texture of the prostate gland. This specifically includes:

1. Resectional or Enucleation procedures: such as transurethral resection of the prostate (TURP), holmium laser enucleation (HoLEP), or simple prostatectomy, etc.
2. Ablative or Vaporization therapies: such as photoselective vaporization (PVP) or water vapor therapy (Rezum), etc.
3. Mechanical implants: such as prostatic urethral lift (UroLift), etc.

Bewick, V., L. Cheek, and J. Ball

2005 Statistics review 14: Logistic regression. Crit Care 9(1):112-8.

Breiman, Leo

2001 Random Forests. Machine Learning 45(1):5-32.

Chen, Tianqi, and Carlos Guestrin

2016 XGBoost: A Scalable Tree Boosting System. Knowledge Discovery and Data Mining, 2016.

Cortes, Corinna, and Vladimir Vapnik

1995 Support-vector networks. Machine Learning 20(3):273-297.

Schonlau, Matthias

2023 The Naive Bayes Classifier. *In* Applied Statistical Learning: With Case Studies in Stata. M. Schonlau, ed. Pp. 143-160. Cham: Springer International Publishing.

Song, Y. Y., and Y. Lu

2015 Decision tree methods: applications for classification and prediction. Shanghai Arch Psychiatry 27(2):130-5.

Zhang, Z.

2016 Introduction to machine learning: k-nearest neighbors. Ann Transl Med 4(11):218.

**
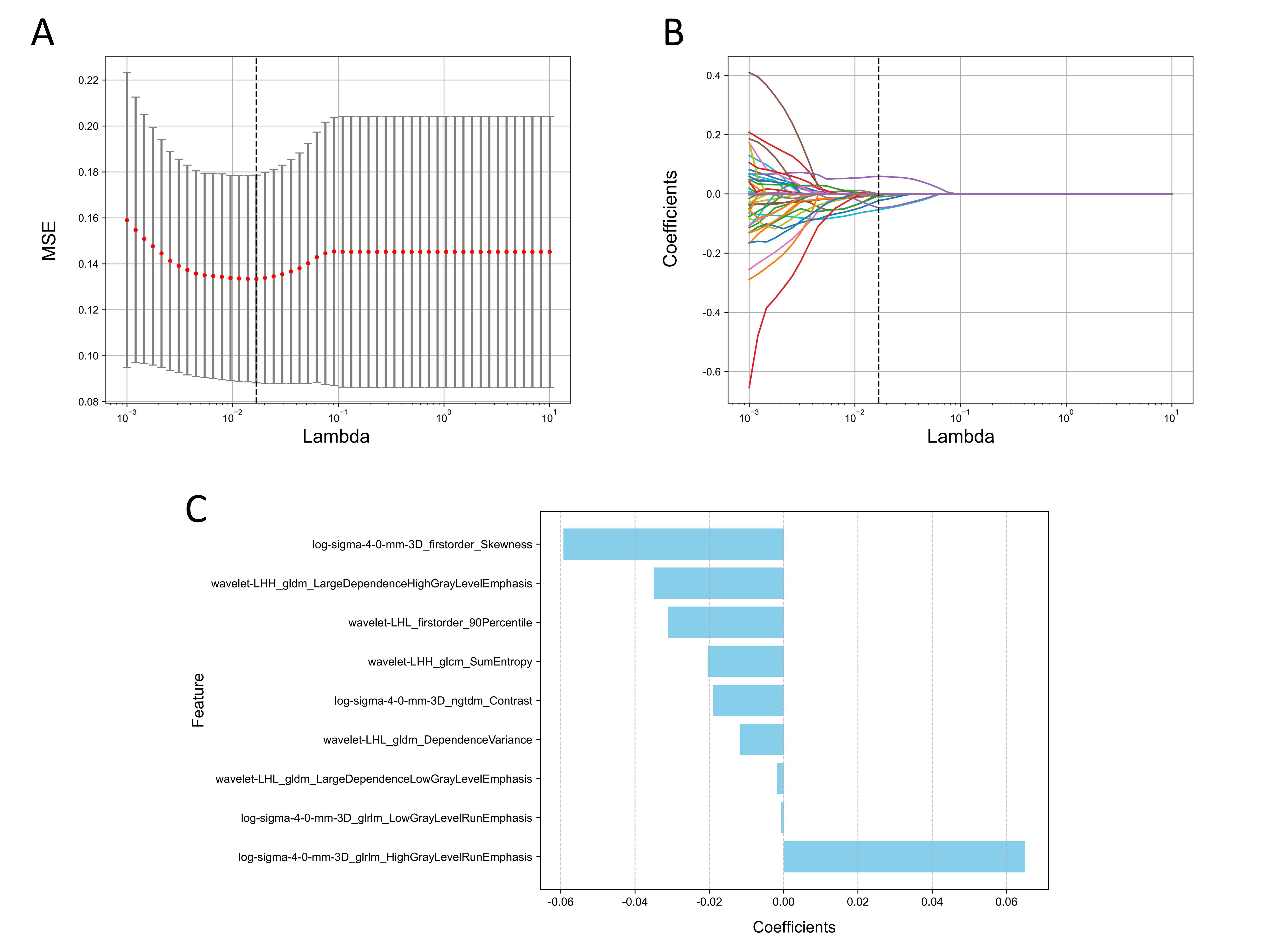
**

**Supplement Figure 1. LASSO regression for radiomics feature selection.**(A) Cross-validation curve for selecting the optimal lambda value.
(B) Coefficient profiles of radiomics features as lambda changes.
(C) The most important radiomics features and their weights selected by LASSO.

**
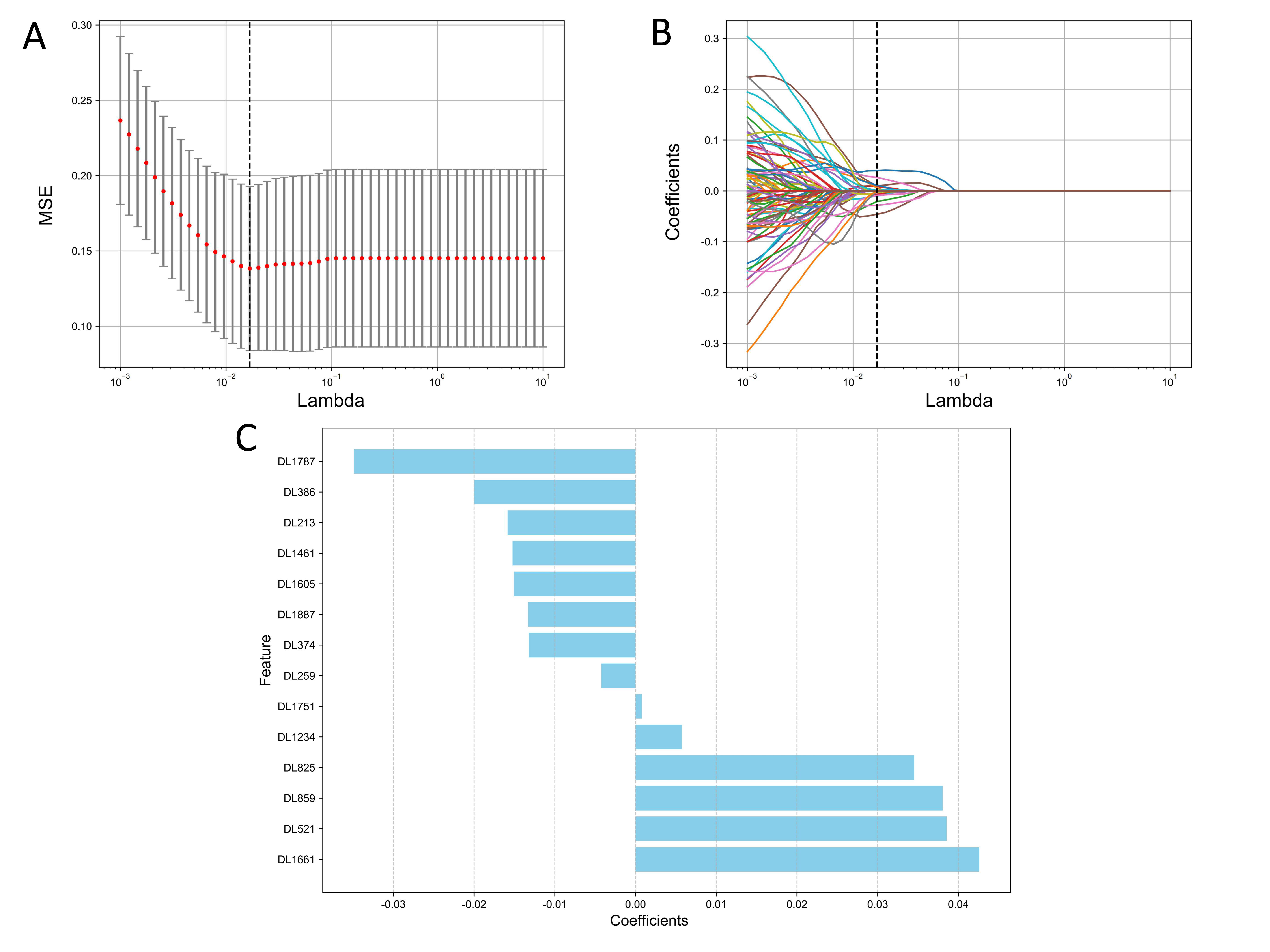
**

**Supplement Figure 2. LASSO regression for selection of deep learning features.**(A) Cross-validation curve showing the optimal lambda value.
(B) Coefficient profiles of deep learning features as lambda changes.
(C) The selected deep learning features and their weights determined by LASSO.

**
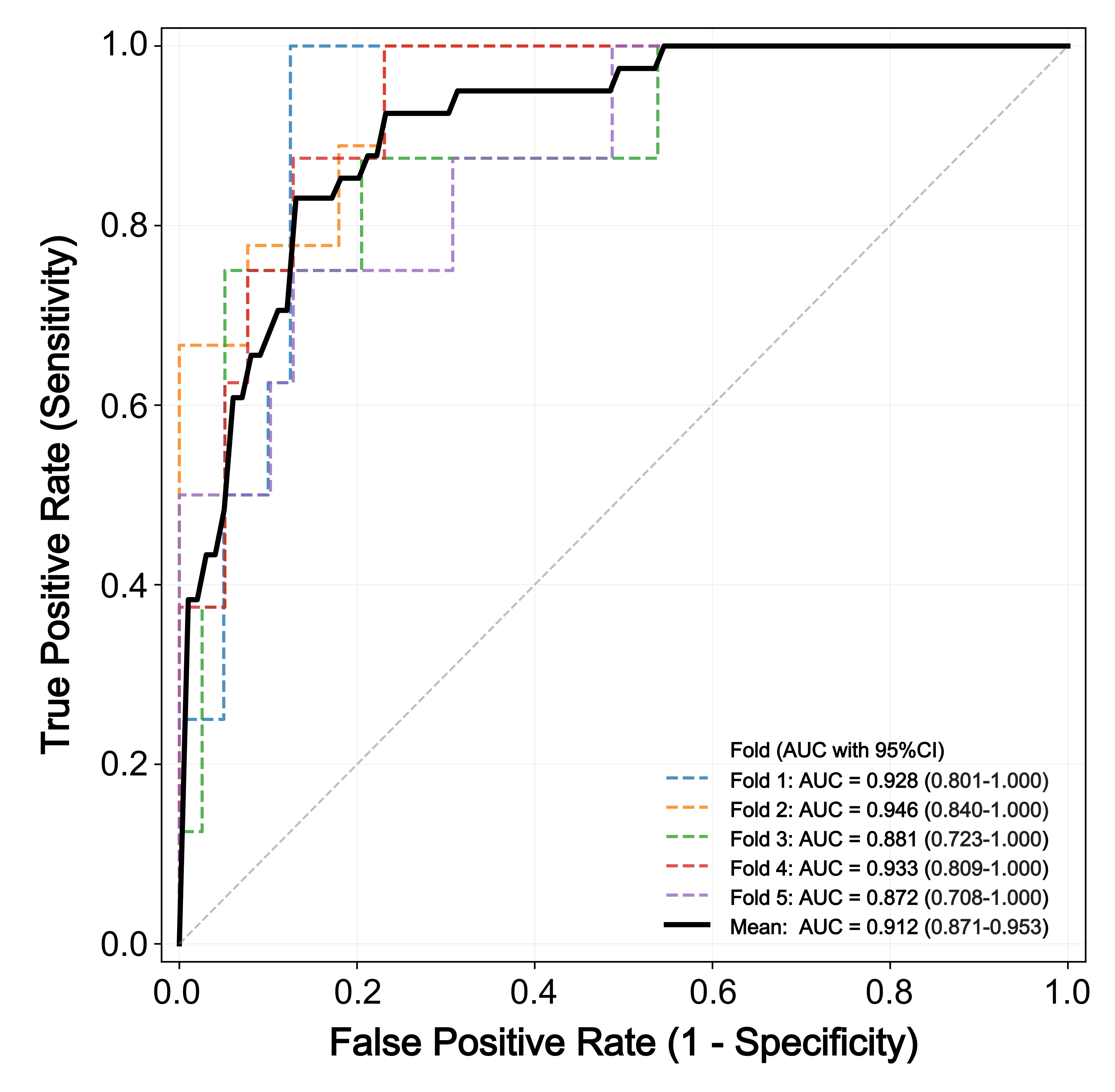
**

**Supplement Figure 3. Five-fold cross-validation of DRBN in the training cohort.**

**
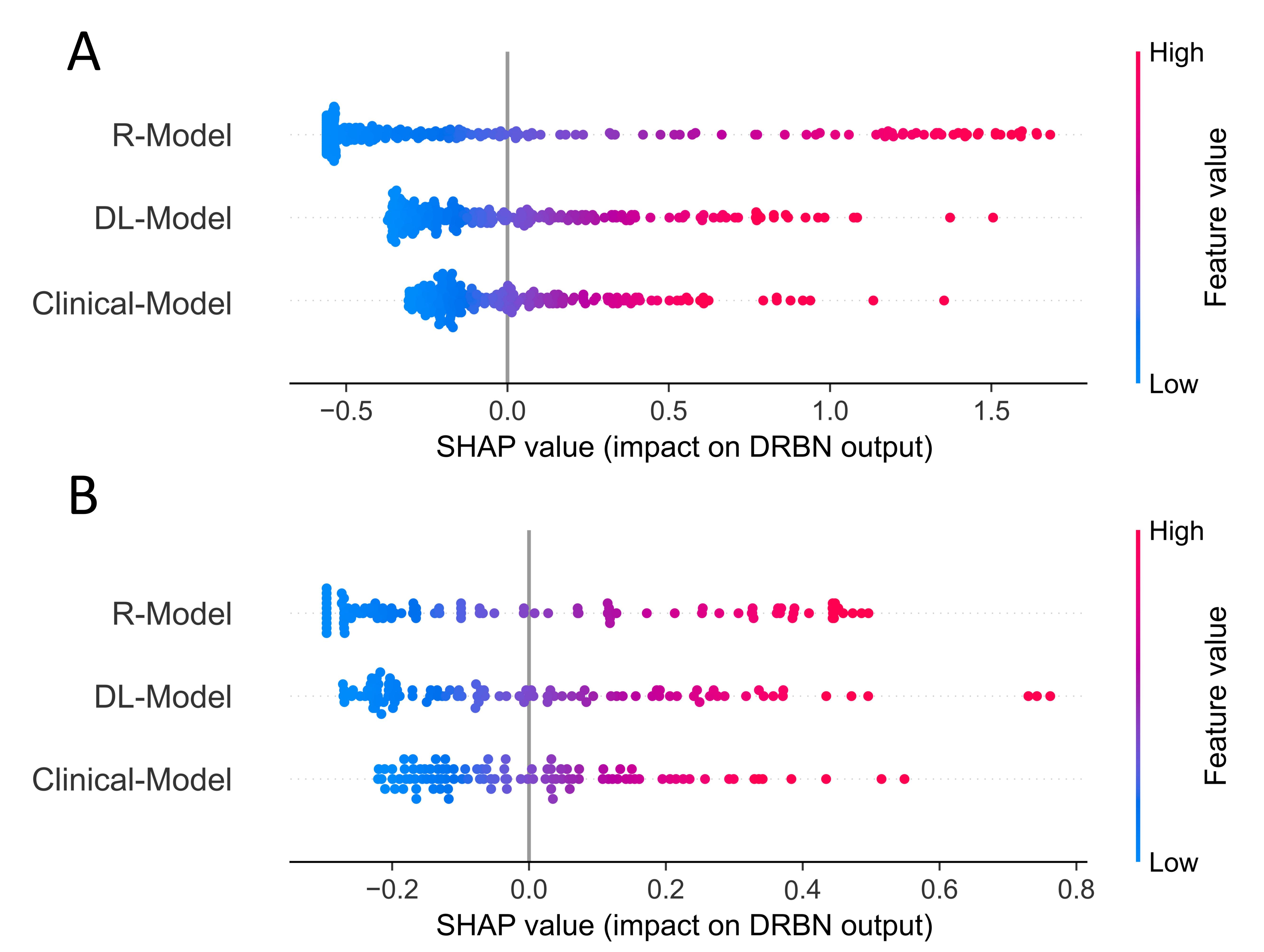
**

**Supplementary Figure 4. SHAP beeswarm plots for the DRBN model.**

(A-B) SHAP analysis illustrating the feature importance within the DRBN framework in the training cohort (A) and validation cohort (B). The R-model exhibited the highest mean SHAP value, indicating its dominant contribution to the final predictive output, followed by the DL-model and the clinical model.

| **Supplementary Table 1. Main Sequence Parameters of Prostate MRI Scans** | | | | |
| --- | --- | --- | --- | --- |
| Parameter | T1WI-TRA | T2WI-TRA | T2WI-SAG | T2WI-COR |
| TR (ms) | 841 | 4559 | 3661 | 4003 |
| TE (ms) | 8.6 | 110 | 110 | 110 |
| FOV (mm) | 400×400 | 240×240 | 240×240 | 240×240 |
| Matrix | 320×224 | 320×224 | 320×224 | 320×224 |
| Slice thickness (mm) | 3 | 3 | 4 | 4 |
| Slice gap (mm) | 1 | 1 | 1 | 1 |
| NEX | 1 | 2 | 2 | 2 |
| Flip angle (°) | 111 | 111 | 111 | 111 |
| Bandwidth | 41.67 kHz | 41.67 kHz | 31.25 kHz | 31.25 kHz |
| Abbreviations: TR, repetition time; TE, echo time; FOV, field of view; NEX, number of excitations. | | | | |
